# In this issue of the *Korean Journal of Women Health Nursing* September 2020

**DOI:** 10.4069/kjwhn.2020.09.23

**Published:** 2020-09-29

**Authors:** Sue Kim

**Affiliations:** College of Nursing, Yonsei University, Seoul, Korea

Greetings to our avid readers. September brings us seasonal changes and a sense of contemplation as we count the slim pages left on the calendar.

Our issue article offers inspiration and insight as a thoughtful reflection on 2020, the International Year of the Nurse and Midwife. The invitation to uncover our nursing stories and share our collective voice has already gained more than 1,500 views on KJWHN’s online website. The Korean Society for Women Health Nursing also responded to the author’s nudge, by putting out a call for nurses and nursing students to submit written and/or artistic expressions of their stories. Please join us for the December issue for a glimpse of selected works.

On another note, September also signaled the start of a new academic semester and accreditation season for nursing schools in Korea. In addition to coronavirus disease 2019 (COVID-19) dragging on, I suspect these factors may have affected submissions and a slower review process. Although the number of articles has decreased, we trust you will find the variety of topic clusters informative.

• ***Midwifery role development:*** An English article on professionalism and job satisfaction among Korean midwives according to their practice site, highlights midwives’ current limitations and discusses solutions.

• ***Reproductive health:*** Nursing students are often exposed to stress and fatigue and a study examined factors affecting premenstrual symptoms in this particular group.

• ***Maternal health:*** A study explored the narratives of preterm birth experiences through text network analysis. Readers may find it interesting to see how MAXQDA software (VERBI Software, Berlin, Germany) visualized and aided identification of clinical symptom expressions.

• ***Nursing education:*** A study tested a labor care high-fidelity simulation program for nursing students who had not yet experienced clinical practicum. With on-site direct learning an ongoing challenge due to COVID-19, the authors’ report on its effects on knowledge, critical thinking, and clinical performance, may be helpful for nurse educators.

• ***Women’s mental health:*** A study investigated suicide ideation among female late adolescents in rural areas, an underserved group. Affective factors and personal experiences were found as key.

We hope this issue will inspire you to share your nursing stories (and manuscripts) with others.

